# ANKLE1 cleaves mitochondrial DNA and contributes to cancer risk by promoting apoptosis resistance and metabolic dysregulation

**DOI:** 10.1038/s42003-023-04611-w

**Published:** 2023-03-01

**Authors:** Piotr Przanowski, Róża K. Przanowska, Michael J. Guertin

**Affiliations:** 1 Department of Biochemistry and Molecular Genetics, University of Virginia School of Medicine, Charlottesville, VA USA; 2 Department of Biomedical Engineering, University of Virginia School of Medicine, Charlottesville, VA USA; 3 Center for Cell Analysis and Modeling, University of Connecticut, Farmington, CT USA; 4 Department of Genetics and Genome Sciences, University of Connecticut, Farmington, CT USA

**Keywords:** Mechanisms of disease, Cancer epidemiology

## Abstract

Alleles within the chr19p13.1 locus are associated with increased risk of both ovarian and breast cancer and increased expression of the *ANKLE1* gene. ANKLE1 is molecularly characterized as an endonuclease that efficiently cuts branched DNA and shuttles between the nucleus and cytoplasm. However, the role of ANKLE1 in mammalian development and homeostasis remains unknown. In normal development ANKLE1 expression is limited to the erythroblast lineage and we found that ANKLE1’s role is to cleave the mitochondrial genome during erythropoiesis. We show that ectopic expression of ANKLE1 in breast epithelial-derived cells leads to genome instability and mitochondrial DNA (mtDNA) cleavage. mtDNA degradation then leads to mitophagy and causes a shift from oxidative phosphorylation to glycolysis (Warburg effect). Moreover, mtDNA degradation activates STAT1 and expression of epithelial-mesenchymal transition (EMT) genes. Reduction in mitochondrial content contributes to apoptosis resistance, which may allow precancerous cells to avoid apoptotic checkpoints and proliferate. These findings provide evidence that ANKLE1 is the causal cancer susceptibility gene in the chr19p13.1 locus and describe mechanisms by which higher ANKLE1 expression promotes cancer risk.

## Abstract (web summary)

Ankyrin repeat and LEM-domain containing protein 1 (ANKLE1) cleaves the mitochondrial genome during erythropoiesis and ectopic expression of ANKLE1 promotes genome instability and apoptotic resistance, which contributes to breast cancer risk.

## Introduction

Alleles within the cancer susceptibility locus chr19p13.1 were first found to modify the risk of breast cancer in BRCA1 mutation carriers, triple negative breast cancer (TNBC), and ovarian cancer^1,2^. The authors suggested that *BABAM1* was acting as the causal gene to modify breast and ovarian cancer risk based upon its physical interaction with the BRCA1 protein complex and its expression in ovarian cancer. Integration of GWAS data with expression quantitative trait loci (eQTL) analysis implicated either *ABHD8* and/or Ankyrin repeat and LEM-domain containing protein 1 (*ANKLE1*) as candidate causal genes^3^. The authors concluded that *ABHD8* was the most plausible causal gene in the locus based upon chromatin conformation assays, deletion analysis, expression data, and cell migration experiments^3^. Later work integrated GWAS and eQTL analysis, along with evolutionary conservation data, ChIP-seq, and chromatin accessibility data to identify the likely causal variant and causal gene, with a focus on variants that disrupt transcription factor binding^4^. This work also proposed that a variant within a CCCTC-binding factor (CTCF) binding site reduces the affinity for CTCF and causes *ANKLE1* expression to increase^4^. This work suggested that *ANKLE1* was the most likely the casual gene within the chr19p13.1 breast and ovarian cancer susceptibility locus.

Few studies have explored the molecular, cellular, and physiological functions of ANKLE1. ANKLE1 was first studied in 2012 as an uncharacterized endonuclease that requires its LEM domain and GIY-YIG motifs for DNA cleavage in vivo^5^. Interestingly, ANKLE1 has high specificity for cleaving branched DNA^6^. Specificity for branched DNA is consistent with an observation in *C.elegans* that its homolog resolves chromatin bridges during late mitosis^7^ and is involved in the the regulation of meiotic recombination repair and chromosome segregation^8^. However, ANKLE1 is dispensable for resolving chromatin bridges, meiotic recombination, and DNA repair in mice^9^. *ANKLE1* is primarily expressed in hematopoietic tissues of vertebrates, but *ANKLE1*-deficient mice are viable without any detectable phenotype in hematopoiesis^9^.

Herein we confirm that ANKLE1 expression is normally limited to the erythroblast lineage and we determine that the developmental role of ANKLE1 is to cleave the mitochondrial genome during erythropoiesis. Although this appears to be the molecular and cellular function of ANKLE1, the biological relevance of this function with regards to organismal development remains unclear. We also explore the undesired role that ectopic expression of ANKLE1 plays in conferring breast cancer risk. We find that ectopic expression of ANKLE1 in epithelial breast cells leads to genome instability, the Warburg effect, and resistance to apoptosis.

## Results

### ANKLE1 is the causal gene for breast and ovarian cancer risk in the chr19p13.1 region

**Fig. 1:**
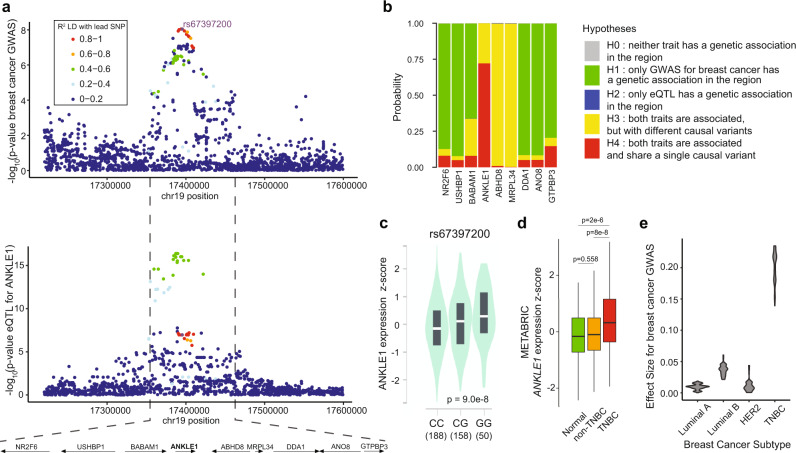
ANKLE1 is the most likely causal gene for Triple-Negative Breast Cancer risk in the 19p13.1 region. **a** The breast cancer susceptibility GWAS variants (upper panel) and ANKLE1 eQTL variants (lower panel) colocalize. **b** Genetic colocalization analysis indicates that both ANKLE1 expression and breast cancer GWAS are associated and likely share a single causal variant. **c** The G allele of rs67397200, which is associated with increased breast and ovarian cancer risk, is associated with higher expression of ANKLE1 in breast tissue. The number of individuals with each genotype is indicated in parentheses. **d** ANKLE1 expression as measured by METABRIC is higher in Triple Negative Breast Cancer (TNBC) than in Normal (tumor-adjacent) or non-TNBC (Luminal A, Luminal B, HER2) tissue (*p*-values are calculated with a two-tailed t-test). **e** Violin plots illustrate that TNBC is the predominant breast cancer subtype associated with the alleles within the 19p13.1 locus.

Expression quantitative trait loci (eQTL) data have revolutionized how geneticists identify candidate causal genes from genome-wide association study (GWAS) loci. We integrated the most recent meta-analysis of breast cancer GWAS^10^ and Genotype-Tissue Expression (GTEx) project data; we found that *ANKLE1* eQTL variants colocalize with the cancer susceptibility GWAS variants (Fig. 1a). In contrast, we found minimal colocalization with other genes in the region (Fig. S1A). Statistical colocalization analysis^11^ demonstrates that *ANKLE1* expression and genetic risk of breast cancer share a single casual variant in the locus with a probability of 0.75 (Fig. 1b). This finding is consistent with recent integrative genomic analyses^4,12,13^. Alleles that are associated with increased risk of breast and ovarian cancer are associated with high expression of *ANKLE1* (Fig. 1c and Fig. S1B). *ANKLE1* is more highly expressed in triple negative breast cancers (TNBC) compared to adjacent normal tissue and other breast cancer subtypes (Fig. 1d). Although we do not claim to identify the causal variant(s) that lead to increased cancer risk, these analyses confirm that ANKLE1 is the most likely causal gene within the region.

Breast cancer is classified into at least four distinct subtypes. The chr19p13.1 locus was originally identified as a breast cancer risk region in BRCA1 mutation carriers and TNBC patients. We compared the effect sizes for the most significant polymorphisms in this region and confirmed that the variants specifically contribute to risk of developing TNBC (Fig. 1e). We also compared eQTL significance to disease risk effect size for all genes in the region and these values are most highly correlated with the *ANKLE1* eQTL in TNBC (Fig. S1C).

### Expression of ANKLE1 in normal tissue is limited to erythroblasts

**Fig. 2:**
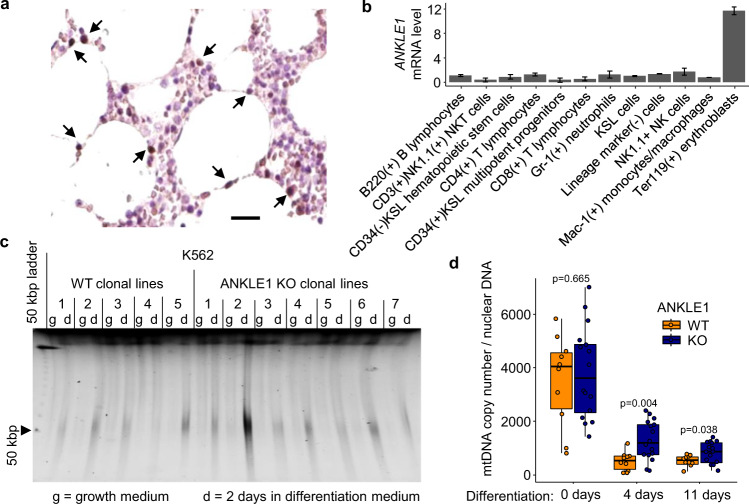
The role of ANKLE1 in development is limited to mtDNA degradation in differentiating erythroblasts. **a** Immunohistochemistry (brown) for ANKLE1 in human bone marrow tissue highlights rare population of cells expressing ANKLE1, as shown by the arrows. Images were reproduced with permission from https://www.proteinatlas.org/ENSG00000160117-ANKLE1/tissue/bone+marrow#img. The scale bar is 20 μm. **b** Microarray data (GDS3997, probe 1443978at ANKLE1) from human bone marrow shows that ANKLE1 is specifically expressed in the erythroblast lineage^10^. **c** Nuclear DNA fragmentation is no different between wild-type (WT) and knock-out (KO) ANKLE1 K562 cells, as shown by pulse field gel electrophoresis (PFGE) analysis of DNA isolated from undifferentiated and differentiated (2 days) K562 cells. **d** Quantitative PCR with primers specific to nuclear and mitochondrial DNA shows that mitochondrial DNA copy number is higher in ANKLE1 KO K562 clonal lines compared to WT lines during differentiation (*p*-values are calculated with a two-tailed t-test).

Although *ANKLE1* gene expression is associated with breast and ovarian cancer, no Mendelian inherited human diseases are caused by *ANKLE1* mutations and *ANKLE1* knockout mice have no discernible phenotypes^9^. In an effort to understand the normal biological role of ANKLE1, we examined ANKLE1 protein expression in different tissues in the Human Protein Atlas^14^. ANKLE1 protein is only detected in a small subpopulation of cells within the bone marrow tissue (Fig. S2A and Fig. 2a). Expression analysis of RNA isolated from different bone marrow cell subpopulations indicates that ANKLE1 expression is limited to erythroblasts (Fig. 2b)^15^. *ANKLE1*’s expression increases during erythroblast differentiation (Fig. S2B), which indicates a possible role in red blood cell development. We hypothesized that ANKLE1 promotes enucleation and DNA fragmentation in erythrocytes based on its expression and the presence of its endonuclease domain. We leveraged an in vitro model of erythroblast differentiation of human leukemia K562 cells to test this hypothesis^16,17^. Consistent with the expression pattern observed in human erythroblast differentiation (Fig. S2B)^18^, *ANKLE1* is transcriptionally activated in differentiating K562 cells (Fig. S2C). Contrary to our expectations, DNA fragmentation is indistinguishable throughout differentiation between clonal *ANKLE1* knockout (KO) and clonal wild-type (WT) K562 cells (Fig. 2c & Fig. S2E, I). This result is consistent with earlier experiments showing that ANKLE1 is dispensable for normal erythropoiesis in *ANKLE1* KO mice^9^. Lastly, differentiating *ANKLE1* KO clones enucleate at a slightly faster rate (Fig. S2F) and contain normal hemoglobin levels (Fig. S2G).

### ANKLE1 localizes to the mitochondria to promote mtDNA degradation and mitophagy

In addition to removing their nucleus, erythrocytes also digest their mitochondria and degrade mtDNA. Mitochondrial DNA is circular, but it is also distinct from nuclear DNA with respect to the lack of nucleosomes and a unique structural feature known as a displacement loop (D-loop)^19^. The D-loop structure contains two branched DNA sites; ANKLE1 cleaves branched DNA orders of magnitude more efficiently than B-form DNA^6^. We hypothesized that ANKLE1 functions to cleave mitochondrial DNA in red blood cell development to facilitate mtDNA degradation. We found that mitochondrial DNA copy number is higher in *ANKLE1* KO lines throughout differentiation (Fig. 2d). Recall that enucleation increases from 13% to 20% of cells on day 4 of differentiation (Fig. S2F), but mitochondrial DNA increases by over two-fold (Fig. 2d). Since we normalize mtDNA based upon the nuclear DNA, we expect an 8% (100% - 80%/87%) increase in mitochondrial DNA levels. This modest difference in nuclear DNA does not explain the two-fold difference in increased mitochondrial DNA content. Mitophagy, or autophagy of the mitochondrial organelle, and enucleation occur during erythropoiesis in humans and in the K562 model^20^ (Fig. S2D). Increased mtDNA is not accompanied by an increase in mitochondrial mass (Fig. S2H), which suggests that mitophagy is not impaired in *ANKLE1* KO cells.

**Fig. 3:**
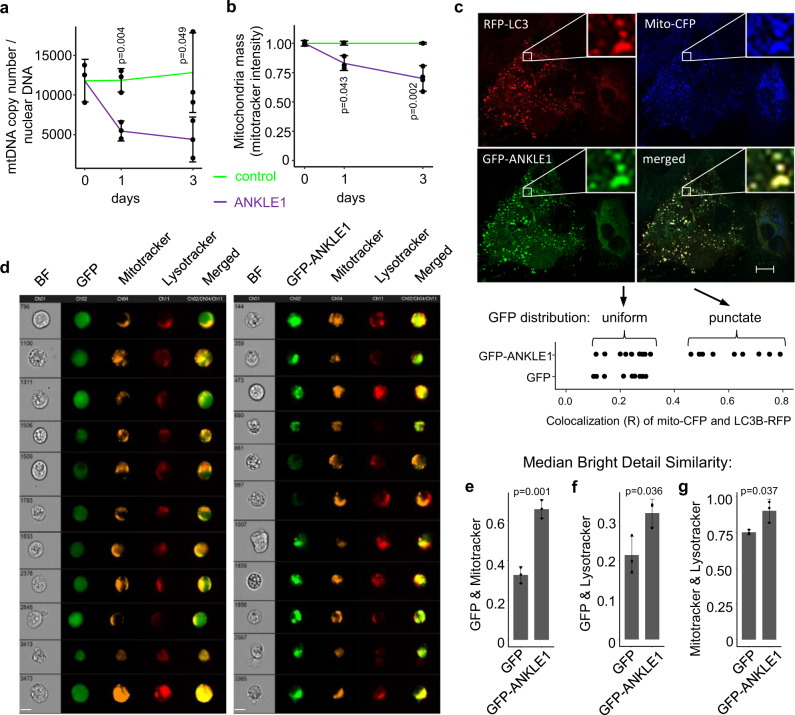
ANKLE1 localizes to mitochondria, degrades mtDNA, and leads to mitophagy. **a**, **b** Overexpression of ANKLE1 decreases mtDNA level (**a**) and decreases mitochondria mass (**b**) in HEK293T cells compared to the GFP expressing controls. **c** Confocal microscopy imaging (scale bar 10 μm) with fluorescent-tagged mitochondrial targeting sequence present in the N-terminus of COX8, ANKLE1, and LC3 proteins shows colocalization of ANKLE1, mitochondria, and autophagasomes when GFP-ANKLE1 form puncta. The lower portion of the panel quantifies this relationship alongside the GFP control from Fig. S3B. **d** Representative cells from imaging flow cytometry of HEK293T cells overexpressing GFP (control) or GFP-ANKLE1, stained with DAPI (to exclude dead cells), mitotracker, and lysotracker (scale bar 10 μm) show colocalization of ANKLE1, mitochondria, and lysosomes. **e**, **f** We quantified all the imaging flow cytometry data with bright detail similarity analysis of GFP, mitotracker, and lysotracker which more rigorously shows that ANKLE1 colocalizes with both mitochondria (**e**) and lysosomes (**f**). **g** The same analysis shows that mitochondria and lysosomes colocalize more in the presence of ANKLE1, indicating an increase in mitophagy (*p*-values are calculated with a two-tailed t-test).

The *ANKLE1* knockout model of erythropoiesis, mtDNA quantification data, and the preferential cleavage of branched DNA all suggest a role for ANKLE1 in regulating mtDNA abundance through D-loop cleavage. We directly characterized the phenotypes associated with overexpression of ANKLE1 in HEK293T cells. We sorted cells to select for non-apoptotic cells that express either GFP-ANKLE1 or GFP alone (Fig. S3A). We found that ANKLE1 expression reduced the levels of mtDNA by over two-fold (Fig. 3a) and decreased mitochondrial mass by 25% (Fig. 3b). Next, we aimed to determine whether ANKLE1 induces mitophagy by quantifying the colocalization of the autophagasome protein marker LC3 and mitochondria, which is indicative of active mitophagy. We used confocal microscopy to show that mitochondria and autophagasomes stained by either Mito-CFP (an N-terminal mitochondrial localization peptide from COX8) and RFP-LC3 colocalize (Fig. 3c). The control GFP expressing cells do not form punctate distributions that colocalize with autophagasomes and mitochondria (Fig. 3c and Fig. S3B). Moreover, ANKLE1 also colocalizes with staining of lysosome and mitochondria organelles as measures by lysotracker and mitotracker (Figure S3C). The colocalization analyses suggest a mitophagy mediates the decrease in mitochondrial mass, so we blocked autophagasome/lysosome fusion with Bafilomycin A1 to confirm the direct role of autophagy. Bafilomycin A1 inhibits normal turnover of the mitochondria in the control cells and abolishes the effect of ANKLE1-induced decrease in mitochondrial mass (Fig. S3D). We further quantified mitophagy by imaging flow cytometry of cells with either GFP or GFP-ANKLE1 that were stained with mitotracker, lysotracker and DAPI (Fig. 3d). Bright detail similarity analysis confirmed that ANKLE1 colocalizes with mitochondria (Fig. 3e) and, to smaller extent, with lysosomes (Fig. 3f). We found that ANKLE1 is uniformly distributed in cytoplasm in some cells, so we compared these cells to cells characterized by punctate ANKLE1 distribution. Cells with punctate distribution of ANKLE1 have reduced mitochondrial mass (Fig. S3E, F), which suggests that ANKLE1 translocates to the mitochondria and precipitates mitochondrial degradation. Lastly, mitochondria colocalize with lysosomes more frequently in the presence of ANKLE1, indicating that mitophagy is increased in GFP-ANKLE1 cells compared to GFP-alone (Fig. 3g).

### Genomic association studies and eQTL data reveal that ANKLE1 expression inversely correlates with mitochondrial DNA copy number

**Fig. 4:**
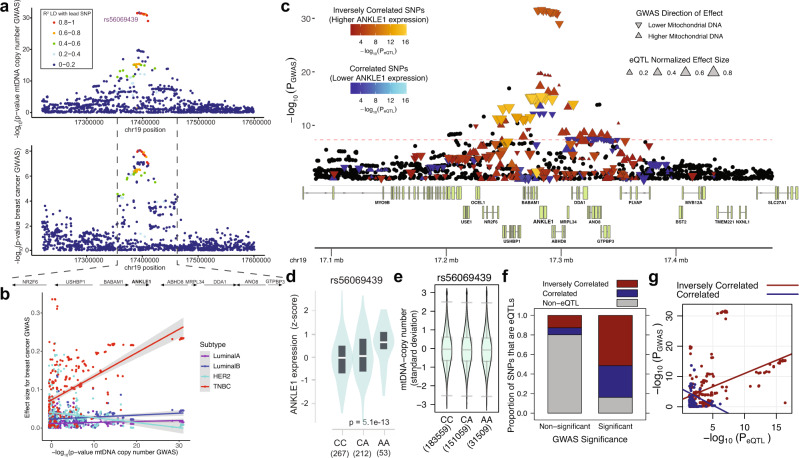
ANKLE1 expression inversely correlates with mitochondrial DNA copy number. **a** Locus Zoom plots for mtDNA copy number and breast cancer risk show similar structure with a common linkage block for both phenotypes. Colocalization analysis indicates that mtDNA copy number and breast cancer risk likely share a single causal allele with 99% probability as measured by the coloc package^11^. **b** The relationship between breast cancer risk effect size and mtDNA-CN significance is specific to the TNBC breast cancer subtype. **c** Integrative analysis of eQTL data from GTEx^12^ and mtDNA copy number GWAS^13^ show that the GWAS alleles associated with lower mtDNA are associated with higher ANKLE1 expression^14^. **d** The lead SNP for mtDNA copy number is an eQTL for ANKLE1. The minor allele associates with higher ANKLE1 expression^12^. **e** The minor rs56069439 allele associates with reduced mitochondrial DNA copy number^13^. **f** Over 50% of the genome-wide significant GWAS variants for mtDNA correlate with ANKLE1 eQTL in the expected direction (higher ANKLE1 associates with lower mtDNA). **g** A scatter plot of log-transformed *p*-values from the mtDNA GWAS versus log-transformed p-values from the ANKLE1 eQTL data shows evidence of colocalization.

Independent studies found that variants within the chr19p13.1 locus associate with DNA copy number (mtDNA-CN) in human blood^21–23^, which is consistent with a role of ANKLE1 in regulating mtDNA copy number in erythropoiesis. The same haplotype block that associates with mtDNA-CN is the lead linkage block that associates with breast cancer risk (Fig. 4a). Just as ANKLE1 is more highly expressed in TNBC cancers and the effect size for the breast cancer GWAS is amplified in TNBCs (Fig. 1d, e), the association between mtDNA-CN and breast cancer risk effect size is unique to the TNBC subtype (Fig. 4b). This is consistent with previous observations that TNBC tumors display a higher frequency of mitochondrial defects and lower mtDNA-CN compared to other breast cancer subtypes^24^.

Colocalization analysis indicates that the most significant mtDNA-CN GWAS variants are also eQTLs for *ANKLE1* (Fig. 4c); moreover, the direction of effect is consistent with a role of ANKLE1 in digesting mitochondrial DNA. Higher *ANKLE1* expression alleles (Fig. 4d) associate with lower mtDNA-CN (Fig. 4e)^23^. The majority of genome-wide significant alleles have an inverse correlation relationship between *ANKLE1* expression and mtDNA-CN (Fig. 4f). The –log_10_ *p*-values for *ANKLE1* expression (eQTL) and mtDNA-CN (GWAS) are correlated exclusively for variants that display an inverse relationship between *ANKLE1* expression and mtDNA-CN (Fig. 4g). Mitochondrial DNA is depleted in breast cancer tissue relative to normal tissue^25^. These findings are consistent with the proposed role of ANKLE1, which is to decrease the level of mtDNA in human cells. Taken together these integrative genomics analyses suggest that susceptibility to breast cancer, ANKLE1 expression, and mtDNA-CN phenotypes share common causal genetic variant(s) within the chr19p13.1 region.

### Expression of ANKLE1 leads to mitochondria degradation, DNA damage, Epithelial to Mesenchymal transition, and STAT1 activation

**Fig. 5:**
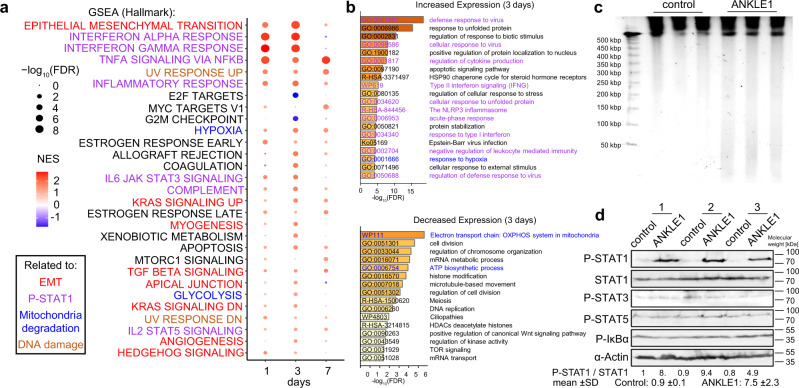
Expression of ANKLE1 leads to STAT1 activation, mitochondria degradation, DNA damage, and Epithelial to Mesenchymal transition. **a** Gene Set Enrichment Analysis (GSEA) of RNA-seq results for ANKLE1 overexpressing cells versus control cells identifies hallmarks of STAT1 activation, mitochondria degradation, DNA damage, and Epithelial to Mesenchymal transition. **b** Gene ontology analysis for differentially expressed genes (Fig. S5C) after 3-days post-transfection of ANKLE1 cells versus control cells show enrichment for STAT1 activation and mitochondria degradation. **c** ANKLE1 cuts nuclear DNA in non-apoptotic cells, as shown by DNA Pulse Field Gel Electrophoresis (PFGE) from non-apoptotic cells that overexpress ANKLE1 (24 h post-transfection). **d** ANKLE1 leads to STAT1 activation after 24 h of ANKLE1-transfection, as shown by western blots probed for P-STAT1, STAT1, P-STAT3,P-STAT5, P-IκB and α-Actin of three independent replicates per condition. The mean and standard deviations for the control and ANKLE1 are shown below the blot. The *p*-value for differential phosphorylation as measured by a two-tailed T-test is 0.008.

Since the molecular biology and genetic data indicate that ANKLE1 function is mediated through the mitochondria in normal and disease states, we hypothesized that ANKLE1 overexpression would lead to changes in expression of energy production and respiration genes. We performed genomic transcriptome profiling (RNA-seq) after overexpression of ANKLE1 for 1, 3, and 7 days (Fig. S5A, C). Several gene set and gene ontology terms related to metabolic changes and mitochondrial regulation are enriched in the differentially expressed genes (Fig. 5a, b and Fig. S5D, E). *Hypoxia* and *glycolysis* are enriched gene sets among the activated genes (Fig. 5a and Fig. S5B). *Electron transport chain: OXPHOS system in mitochondria* is the most significant ontology term for repressed genes (Fig. 5b).

Previous work found that despite harboring endonuclease activity and both a nuclear import and export signal, ANKLE1 is not nuclearly localized and does not cause an extensive DNA-damage response^5,26^. However, we observed that DNA damage response gene sets are activated upon ANKLE1 overexpression (Fig. 5a and Fig. S5B, D). We used a more sensitive assay, Pulse Field Gel Electrophoresis (PFGE), to assay ANKLE1’s ability to cut nuclear DNA when over-expressed. We excluded DNA breaks caused by apoptosis by selecting non-apoptotic cells (Fig. S3A). We found that ANKLE1 cuts DNA with low frequency, resulting in fragments between 50 kbp and 200 kbp (Fig. 5c).

Epithelial-mesenchymal transition (EMT) is the most significantly enriched gene set among ANKLE1 regulated genes (Fig. 5a and Fig. S5B). EMT is also a defining feature of breast cancer transformation^27,28^. Several enriched gene sets represent hallmarks of EMT, such as TGF beta signaling, apical junction, and KRAS signaling (Fig. 5a).

We found that many gene sets that relate to STAT1 signaling were enriched among the gene sets (Fig. 5a, b and Fig. S5B, D, E). Previous work found that mtDNA double strand breaks leads to phosphorylation of STAT1, which leads to genomic transcriptional changes^29^. Taken together with the mitochondria degradation phenotypes (Fig. 3a, b), we hypothesized that mtDNA cleavage by ANKLE1 leads to STAT1 phosphorylation. Indeed, we found that STAT1 is phosphorylated in ANKLE1-expressing cells and this is not accompanied by activation of STAT3, STAT5, or NF-κB (Fig. 5d). MtDNA damage synergizes with nuclear DNA damage to mount a robust type-I interferon response^29^ and the proteins DDX5 and cGAS are critical components of interferon signaling. cGAS is the major innate immune sensor of pathogenic DNA and can sense nuclear DNA breaks. DDX58 senses mtRNA exposed on the mitochondrial surface after mtDNA damage. We observe activation of DDX58 expression and inhibition of cGAS after ANKLE1 overexpression (Fig. S5F). Taken together with the modest nuclear DNA damage after ANKLE1 overexpression (Fig. 5c), this data is consistent with a mechanism by which mtDNA damage activates STAT1 upon ANKLE1 overexpression.

### ANKLE1 preferentially cleaves mitochondrial DNA

**Fig. 6:**
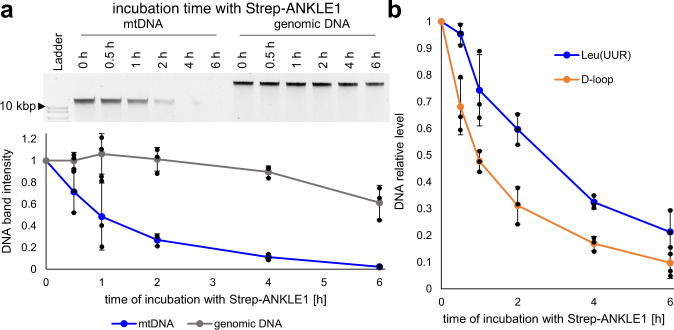
ANKLE1 preferentially cleaves mitochondrial DNA. **a** The top gel is a representative gel of raw data of a time course treatment of purified mtDNA and nuclear DNA with purified ANKLE1. ANKLE1 degrades mtDNA faster than genomic DNA. The lower panel is quantification of three replicates. **b** Quantitative PCR with primers flanking the D-loop or within the tRNA Leu(UUR) gene illustrates that ANKLE1 preferentially cuts the D-loop region of mtDNA. The standard deviation of three independent replicates is shown.

Variants in the chr19p13.1 locus both increase ANKLE1 expression and the risk of developing breast cancer. If ANKLE1 digests mtDNA in these cells, we expect that mtDNA content in breast cancer is inversely correlated with *ANKLE1* expression. We analyzed data that quantified mtDNA content and ANKLE1 expression in tumor samples and matched normal control samples^25^. Individuals with the highest ANKLE1 expression had consistently lower mtDNA content and mtDNA content is inversely correlated with *ANKLE1* expression when all patients are considered together (Fig. S4A). We next sought to determine whether purified ANKLE1 (Fig. S4B) prefers purified mitochondrial DNA as a substrate. We isolated both mtDNA and nuclear DNA and incubated with ANKLE1 and monitored DNA degradation over 6 h. ANKLE1 cleaves both DNA species, but mtDNA is degraded more rapidly and completely over the six hour time course (Fig. 6a). Since previous work established that ANKLE1 preferentially cuts branched DNA^6^, we quantified the degradation of mtDNA with primers that span the D-loop and genic primers. The kinetics of degradation as measured by qPCR mirror the quantification by gel densitometry (Fig. 6b). The D-loop contains brached DNA and consistent with our expectation, the D-loop mtDNA is degraded with faster kinetics and more completely than genic mtDNA (Fig. 6b).

### ANKLE1 expression in normal breast epithelium cells drives the Warburg effect and apoptosis resistance in TP53 mutant cells

**Fig. 7:**
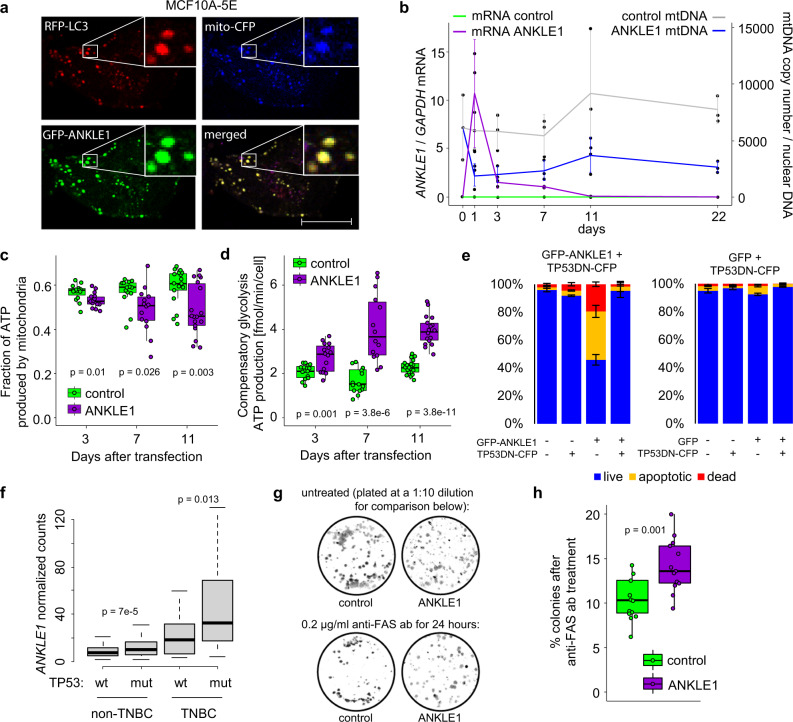
ANKLE1-induced mitophagy in normal breast cancer cells shifts the metabolism to glycolysis and increases resistance to apoptosis in TP53 negative cells. **a** Confocal microscopy images (scale bar = 10 μm) of MCF10A-5E cells overexpressing GFP-ANKLE1, N_terminal_COX8-CFP, and RFP-LC3 proteins shows colocalization of ANKLE1, mitochondria, and autophagasomes. **b** We quantified relative levels of ANKLE1 mRNA and mtDNA over a 22 day time course of transient ANKLE1 expression. Although ANKLE1 levels return to baseline (Fig. S6A), the decrease of mtDNA level is maintained. **c**, **d** We used the Seahorse XF Real-Time ATP Rate Assay to show that ANKLE1 decreases the fraction of ATP produced by mitochondria (**c**) and ANKLE1 increases maximum compensatory glycolysis in MCF10A-5E (**d**). **e** Flow cytometry analysis quantification of Figure S6E shows that ANKLE1 induces apoptosis in MCF10A-5E cells and apoptosis is abolished by a TP53 dominant negative mutant. **f** The level of ANKLE1 mRNA in human noTNBC and TNBC samples isolated from TP53 wild-type or mutant tumors indicates higher expression when TP53 is mutated. **g**, **h** ANKLE1 overexpression produced more colonies when MCF10A-5E cells were treated with anti-FAS activating antibodies, even though ANKLE1 transient expression occurred 11 days prior to anti-FAS treatment (*p*-values are calculated with a two-tailed t-test).

Recall that ANKLE1 expressing cells exhibit hallmarks of gene expression that indicate a switch from oxidative phosphorylation to glycolysis. In an effort to recapitulate the processes that may lead to transformation of normal breast cells, we began experiments in the MCF10A-5E cell line. MCF10A-5E cells are clonally-derived from non-tumorigenic epithelial MCF10A cells (ATCC). These cells exhibit homogeneous behavior in 3D cultures and express many of the hallmarks of epithelial breast cells, such as epithelial sialomucins, cytokeratins, and milk fat globule antigen^30,31^. Similarly to what we observed in HEK293T cells, ANKLE1 induces mitophagy in MCF10A-5E cells (Fig. 7a). We found transient *ANKLE1* expression leads to chronic phenotypic changes within MCF10A-5E cells. We performed a 22 day time course experiment and we found that *ANKLE1* expression peaks within a day of transfection (Fig. 7b). *ANKLE1* expression gradually decreases to background levels between 11-22 days after transfection (Fig. S6A). mtDNA is decreased to approximately 50% and persists at this level for the remainder of the time course (Fig. 7b). These data indicate that transient *ANKLE1* expression can lead to stable changes in cell metabolism.

We hypothesized that ANKLE1 expression contributes to a decrease in ATP produced by oxidative phosphorylation relative to ATP produced by glycolysis, which is known as the Warburg effect. We simultaneously measured extracellular acidification rate (ECAR) (Fig. S6B) and oxygen consumption rate (OCR) (Fig. S6C) using an ATP rate assay in ANKLE1-transfected and GFP-transfected control MCF10A-5E cells. We found higher acidification and lower oxygen consumption, which reflects lower oxidative phosphorylation and increased glycolysis. ECAR and OCR values were used to directly measure proton efflux rate (see Methods), which is an indirect measure of ATP production by mitochondria. We found that, on average, the fraction of ATP produced by the mitochondria is lower at days 3, 7, and 11 post-transfection (Fig. 7c). Next, we blocked oxidative phosphorylation and quantified ATP produced by glycolysis in ANKLE1 vs. GFP control cells. We found that ANKLE1-transfected cells can produce ATP by glycolysis at a faster rate when oxidative phosphorylation is blocked, this is termed *compensatory glycolysis* (Fig. 7d). We hypothesize that ANKLE1-mediated mitochondria degradation leads to a demand in energy resources that triggers activation of hypoxia (Fig. 5a) and HIF1 pathways (Fig. S5E), which lead to an increase in glycolysis. We repeated these experiments in HEK293T cells and we again found that ANKLE1 reduces the fraction of ATP produced in by the mitochondria and increases compensatory glycolysis (Fig. S6D, E).

Throughout the experiments with ANKLE1 expressing MCF10A-5E cells, we empirically noticed that the fraction of apoptotic cells was higher than what we observed with ANKLE1 overexpression in HEK293T cells. The main genetic difference between these cell lines is the presence of large T antigen that deactivates TP53 in HEK293T cells. MCF10A-5E cells contain active TP53. Taken together with the fact that ANKLE1-mediated risk of breast cancer is primarily through its effect on the TNBC subtype, of which 80% are mutant for TP53^32^, we hypothesized that TP53 mutations allow cells that express ANKLE1 ectopically to evade apoptosis. We tested this hypothesis by transfecting MCF10A-5E cells with vectors carrying either GFP or GFP-ANKLE1 along with a CFP-TP53 dominant negative R175H mutant (TP53DN). We quantified apoptosis after 24 h with AnnexinV-PE and 7-AAD (Fig. S6F). We found that ANKLE1-alone induces apoptosis in 60% of MCF10A-5E cells (Fig. 7e). ANKLE1-driven apoptosis in MCF10A-5E cells is completely abolished in the presence of dominant negative TP53. In contrast, ANKLE1 does not increase apoptosis compared to the GFP control in HEK293T cells (Fig. S6G). If mutant TP53 protects against ANKLE1-induced apoptosis, we would expect that TP53-mutant tumors have higher levels of ANKLE1 expression. We queried the Molecular Taxonomy of Breast Cancer International Consortium (METABRIC) database and we found that breast cancer tissues with mutated TP53 have significantly higher ANKLE1 expression irrespective of their TNBC status (Fig. 7f). We experimentally tested the hypothesis that ANKLE1 overexpression will specifically induce apoptosis in TP53 wild type breast cancer cell lines. We compared ANKLE1-induced apoptosis in TP53 mutant breast cancer cells lines (HCC1806, MDA-MB-231 and MDA-MB-468) to TP53 wild type cells lines (MCF7, HCC1500 and CAL51). ANKLE1 increases apoptosis between 10% and 15% in p53-wild type cell lines and less than 3% in TP53 mutant cell lines (Fig. S6H). These support a model whereby ANKLE1 causes apoptosis in presence of wild-type TP53, and not in presence of mutant TP53. Mitochondria are known to play an essential role in apoptosis by regulating the release of proteins from the intermembrane space, which activates caspases^33^. To further highlight the importance of the mitochondria in apoptosis, previous work showed that modest differences in cellular mitochondrial content in clonal cell lines can determine the apoptotic fate upon activation of death receptor^34^. We designed an experiment to directly test whether ANKLE1-induced depletion of the mitochondria (Fig. 7b) increases the resistance of death receptor-triggered apoptosis in MCF10A-5E cells. Recall that at day 11 ANKLE1 expression is at background levels, but mitochondrial DNA copy number is reduced by 50% (Figure S6A). We found that transiently expressed ANKLE1 prior to day 11 are more resistant to death receptor-induced apoptosis and able to proliferate and form colonies (Fig. 7g, h).

### Transient, ectopic expression of ANKLE1 induces phenotypes consistent with hallmarks of carcinogenesis

**Fig. 8:**
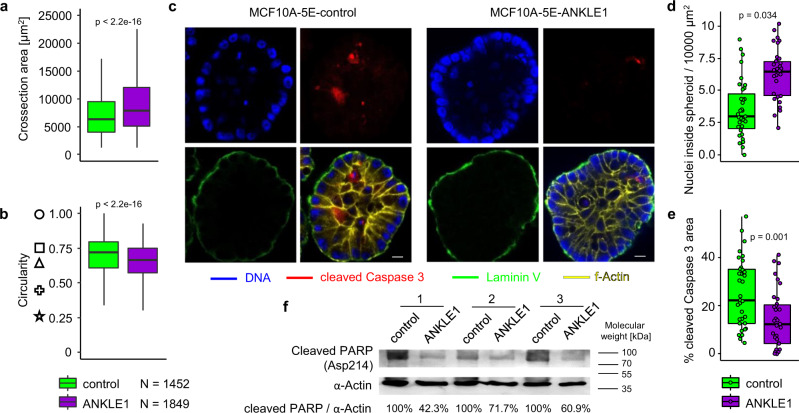
Spheroids formed by normal breast epithelium cells after ANKLE1 expression exhibit precancerous phenotypes. **a** MCF10A-5E cells that transiently expressed ANKLE1 form larger spheroids. **B** MCF10A-5E cells that transiently expressed ANKLE1 form less circular spheroids. Note that the symbols to the left are examples of circularity at the indicated y-value. **c** Representative confocal microscopy images (scale bar = 10 μm) of spheroids stained for DNA, cleaved Caspase 3, and f-Actin reveal the effect of transient ANKLE1 expression. **d**, **e** Spheroids derived from MCF10A-5E cells transiently transfected with ANKLE1 contain more intact nuclei (**d**) and exhibit less cleaved Caspase 3 staining (**e**) inside the spheroid. **f** Western blot shows a lower level of cleaved PARP in protein extracts from spheroids derived from MCF10A-5E cells transiently expressing ANKLE1. We used densitometry to quantify the percent of cleaved PARP relative to the control.

Thus far we have shown that ANKLE1 promotes the Warburg effect and resistance to apoptosis within HEK293T and MCF10A-5E cells. Recall that the MCF10A-5E clone was selected for its ability to form homogenous 3D spheroids on the matrigel surface, which makes the model amenable for measuring features that are associated with transformation and cancer progression. MCF10A-5E cells cultured on matrigel form round hollow spheroids due to apoptosis of internally localized cells and this orderly growth is disrupted upon the introduction of an oncogene^30,35^. We transiently transfected ANKLE1 into MCF10A-5E cells and 11 days later we seeded them onto matrigel for an additional 11 days. We quantified the size and circularity of spheroids with OrganoSeg^36^ (Fig. S7A). Although MCF10A-5E cells no longer express ANKLE1 when they are seeded on matrigel, the spheroids that form are larger (Fig. 8a) and less circular (Fig. 8b and Fig. S7B). We hypothesized that the spheroids that previously expressed ANKLE1 have a defect in apoptosis of internally localized cells, so we stained for cleaved Caspase 3 (a marker of apoptosis) and DNA (Fig. 8c). Indeed, spheroids formed from acute ANKLE1-expressing cells have more internally located intact nuclei (Fig. 8d) and less apoptosis (Fig. 8e). We confirmed less apoptosis in ANKLE1 spheroids by quantifying cleaved PARP protein levels by western blotting (Fig. 8f). These data indicate that transient, ectopic expression of ANKLE1 in breast epithelium provides resistance to apoptosis, which can trigger spheroid phenotypes that are consistent with cancer progression.

## Discussion

**Fig. 9:**
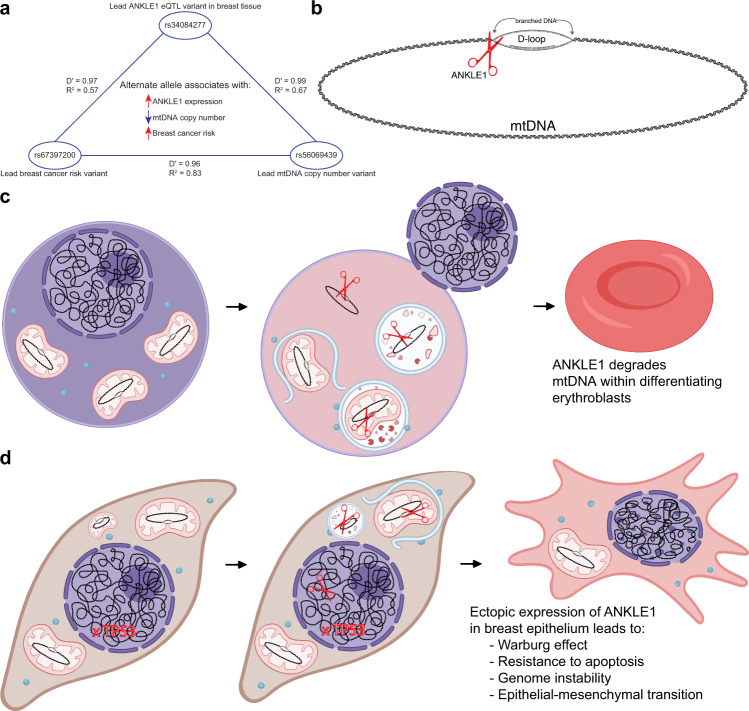
A model of ANKLE1 function in erythroblast differentiation and breast cancer. **a** The lead genetic variants for breast cancer risk, ANKLE1 expression, and mtDNA copy number are genetically linked to one another and each is associated with the other two phenotypes. **b** ANKLE1 preferentially cuts branched DNA and we speculate that ANKLE1-mediated cleavage of the mtDNA D-loop facilitates degradation of mitochondrial DNA. **c** We propose that the role of ANKLE1 in erythroblast differentiation is limited to mtDNA degradation. **d** Ectopic expression of ANKLE1 in breast epithelium also causes mtDNA degradation, and this leads to mitophagy, the Warburg effect and resistance to apoptosis. Additionally, ANKLE1 in the nucleus may contribute to genome instability. Our RNA-seq data indicate that these phenotypes may lead to transcriptional changes that are consistent with the Epithelial-Mesenchymal transition.

Genome-wide association data and expression QTL data suggest common causal variant(s) within the chr19p13.1 locus for breast cancer risk, *ANKLE1* expression, and mtDNA copy number (Fig. 9a). We propose that breast cancer risk variants directly regulate *ANKLE1* expression in breast tissue, which directly leads to mtDNA degradation and results in modified cellular metabolism and homeostasis.

Although there are relatively few reports on the function of ANKLE1, the data suggest that ANKLE1 originally evolved as a means to resolve DNA bridges^7,8^. Resolving DNA bridges is consistent with ANKLE1’s preferred specificity to cleave branched DNA^6^. While the molecular function of cleaving branched DNA remains unchanged in vertebrates, the ANKLE1 protein may have been functionally repurposed for mtDNA degradation in mammals. Mammals have evolved to degrade both their nuclei and mitochondria during erythropoiesis. The nucleus is actively expelled from the cell in a process known as enucleation and mitochondria are digested by mitophagy. Our data support a complementary mechanism to ensure degradation of the mtDNA in differentiating erythroblasts by ANKLE1 (Fig. 2d and Fig. S2H). Although our ANKLE1-GFP overexpression results need to be cautiously interpreted because GFP is not localized to the mitochondria in the control cells, both the ANKLE1 knockout and mtDNA GWAS results^23^ provide independent orthogonal evidence of ANKLE1’s role in mtDNA degradation. The mitochondrial chromosome is circular and more stable because, without free DNA ends, circular DNA is refractory to digestion by exonucleases. Since mtDNA harbors a D-loop with branched DNA structures^19^, *ANKLE1* cleavage of mtDNA may provide a mechanism to linearize the mitochodiral chromosome to ensure its faithful degradation (Fig. 9b). ANKLE1-mediated mtDNA digestion may prevent accumulation of cytosolic mtDNA that escaped from autophagosomes, as this phenomenon is associated with multiple metabolic diseases^37^.

ANKLE1 is not expressed in normal breast tissue and we hypothesize that the ectopic expression of ANKLE1 leads to changes in metabolism, transcription, and cellular homeostasis. In support of this assertion, we show that ANKLE1 triggers cleavage of nuclear DNA in cells without triggering apoptosis (Fig. 5c). The more prominent phenotype we observed is that ANKLE1 induces mtDNA degradation and mitophagy (Fig. 3). The mitochondria phenotypes may drive STAT1 phosphorylation (Fig. 5d) and a metabolic switch from oxidative phosphorylation to glycolysis (Fig. 7).Consistent with these observations, we found modest DNA damage when ANKLE1 is overexpressed, but the most prominent phenotypes that we observed were mitochondrial phenotypes.

In the 1920s Otto Warburg showed that cultured tumor tissues have high rates of glucose uptake and lactate secretion, even in the presence of oxygen (aerobic glycolysis)^38^. Although cancer is caused by mutations within tumor suppressors and oncogenes, the Warburg effect represents a critical metabolic switch for cancer cells to survive and proliferate. There are several postulated roles of the Warburg effect in cancer cells. For instance a higher rate of ATP production by glycolysis may provide a selective advantage when competing for shared and limited energy resources. The Warburg effect has also been proposed to be an adaptation mechanism to support the biosynthetic requirements of uncontrolled proliferation. The increased glucose consumption is used as a carbon source for anabolic processes needed to support cell proliferation, such as the *de novo* generation of nucleotides, lipids, and proteins. As the tumor microenvironment has been more recently studied and appreciated to have a role in cancer biology, it has been proposed that the Warburg effect may present an advantage for cell growth in a relatively acidic and low oxygen microenvironment. Acidification of the microenvironment by lactate may allow for enhanced invasiveness and is a contributor to tumor-associated macrophage polarization that support tumor growth^39^. We suggest that ANKLE1-induced degradation of the mitochondria facilitates the Warburg effect to promote cancer growth.

ANKLE1-induced mitophagy may also promote survival of cancer cells by avoiding apoptosis. We show that ANKLE1 expression leads to lower mitochondria content and resistance to apoptosis caused by death receptor activation (Fig. 7h, i). Activation of the death receptor is the primary mechanism by which the immune system removes dysregulated or mutated cells^40^. In support of this apoptosis-resistance hypothesis, previous work has shown that cells with fewer mitochondria are more resistant to apoptosis induced by death receptor activation^34^. Another possible mechanism by which ectopic ANKLE1 expression may promote tumorigenesis in TP53 mutant cancers, which represent over 80% of TNBCs, is to increase mutational burden. We found that ANKLE1 cuts DNA in the nucleus without causing apoptosis in TP53 mutant cells, which will increase the mutation rate.

Modern genomics and phenotyping can convincingly identify genes that affect a phenotype, in this case ANKLE1 and breast cancer risk. A fundamental challenge is following up with experimental models that accurately recapitulate organismal biology. Alleles such as rs67397200, which affect ANKLE1 expression and contribute to breast cancer risk are common and found at 25% minor allele frequencies. Homozygosity for the risk allele (rs67397200) only affects the relative risk of developing breast cancer with an odds ratio of 1.17. Most GWAS hits have odds ratio in the range of 1.1–1.2. We concede that no model can accurately recapitulate the biology that occurs over 40 years of life that precedes a breast cancer diagnosis. Importantly, if we could recapitulate this biology note that the prevalence of breast cancer is low compared to allele frequency, so the vast majority of individuals with the risk allele do not develop breast cancer. A true model could be deemed a failure because the penetrance of disease incidence is too low. Moreover, ANKLE1 is one of hundreds of genes that contributes to risk of developing cancer, as opposed to a driver like p53, BRCA1, or Ras. Within the field of genetic epidemiology, following up on GWAS candidate genes to identify molecular mechanisms of risk is rate-limiting. With all these limitations in mind, the molecular phenotypes that we observe points to mitochondrial function. Although the system does not perfectly recapitulate the biology of living 40 years with slightly increased levels of ANKLE1 in breast epithelial tissue, the finding that ANKLE1-induces mitophagy in normal breast epithelium cells and shifts the metabolism to glycolysis, while increasing resistance to apoptosis in TP53 negative cells, is consistent with all our other data and models. Likewise, the fact that spheroids formed by normal breast epithelium cells after ANKLE1 expression exhibit precancerous phenotypes is supportive of the model that breast cancer risk attributed by ANKLE1 expression is mediated through ANKLE1’s effect on mitochondria biology.

In summary, the biological role of ANKLE1 is limited to degrading mtDNA in differentiating erythroblasts (Fig. 9c). Ectopic ANKLE1 expression in normal breast cells leads to genome instability and degradation of mtDNA, which causes mitophagy, activation of STAT1, resistance to apoptosis, and a shift to aerobic glycolysis as a means of energy production (Fig. 9d).

## Methods

### Analysis of publicly available data sets and statistical analysis

GWAS data were obtained from published metaanalysis summary statistics^10,23^. Expression QTL data were obtained from GTEx GCS bucket (https://console.cloud.google.com/storage/browser/gtex-resources): dbGaP accession number phs000424.v8.p2^41^. Violin plots of *ANKLE1* expression for different genotypes in breast tissue (Fig. 1 and Fig. 4) were obtained from the GTEx Portal. Colocalization analysis was performed with the coloc and eQTpLot packages^11,42^ using GWAS and QTL p-values to assess colocalization. Analyses we performed under the assumption that a single causal variant is responsible for both the eQTL phenotype and the GWAS phenotype. The effect sizes from the summary statistics were partitioned for different breast cancer subtypes and only the most significant (*p* < 0.0001) GWAS single nucleotide polymorphisms (SNPs) were queried. *ANKLE1* expression in bone marrow subpopulations was analyzed based on microarray data: GDS3997, probe 1443978at^15^. *ANKLE1* expression during erythropoiesis was quantified by RNA-seq (GSE115684)^18^. mtDNA copy number was previously measured by integrating whole exome and whole genome sequencing data^25^. Molecular Taxonomy of Breast Cancer International Consortium (METABRIC) data^43^ were accessed through the Xena platform^44^ and used to obtain TNBC, non-TNBC, normal adjacent, and TP53 mutation status, as well as *ANKLE1* expression (*p*-values were calculated with a two-tailed t-test). We did not reanalyze the METABRIC data and all expression values from METABRIC were previously measured and z-score normalized by the consortium^43^. Circularity was calculated as: 4 π *Area / Perimeter ^2^. All the experiments were performed in at least three independent biological replicates.

### Plasmid construction

The pEGFP-C1-ANKLE1 plasmid that was used for all ANKLE1 expression experiments was kindly provided by Roland Foisner^5^. The pEGFP-C1 plasmid (Clontech V012024) was used as the control. sgRNA constructs for *ANKLE1* knockout were generated by inserting oligonucleotides containing the targeted sequences (5′- TTCAGGGCACAGCCTAGAAC -3′ and 5′- GATTCTGCCCTAGCCCCACC -5′) into the pX458 vector (Addgene Plasmid #48138^45^). Mito-CFP plasmid was obtained from Addgene (Plasmid #58426^46^). LC3-RFP plasmid was obtained from Addgene (Plasmid #21075^47^). TP53DN(R175H)-CFP was constructed by cloning CFP from mitoCFP plasmid into AgeI site of p53 (dominant negative R175H mutant)-pcw107-V5 (Addgene Plasmid #64638^48^), using 5′- GGGTTAGGGATAGGCTTACCACCGGTTTACTTGTACAGCTCGTCCATGC - 5′ and 5′- CTTGTACAAAGTGGTTACCGGAGGATCCGGTGGTGTGAGCAAGGGCGAGGAGCTG - 5′ primers for PCR and In-Fusion Cloning (Takara Bio). The pStrep-ANKLE1 plasmid was obtained by cloning annealed oligonucleotides containing Twin-Strep-tag (5′-CCGGTCACCATGGCGTGGAGCCACCCGCAGTT-CGAGAAAGGTGGAGGTTCCGGAGGTGGATCGG-GAGGTTCGGCGTGGAGCCACCCGC-AGTTCGAAAAAGC 5′ and 5′-GGCCGCTTTTTCGAACTGC-GGGTGGCTCCACGCCGAACCTCCCGAT-CCACCTCCGGAACCTCCACCTTTCTCGAA-CTGCGGGTGGCTCCACGCCATGGTGA 5′) into AgeI, NotI fragment of pEGFP-C1-ANKLE1 to remove GFP.

### Cell culture, treatment, transfection, and generation of stable cell lines

K562, MCF7, CAL-51, HCC1500, MDA-MB-468, HCC1806, MDA-MB-231 and HEK293T cells were purchased from ATCC. MCF10A-5E cells were kindly provided by Kevin Janes. K562 cells were cultured in RPMI supplemented with 10% FBS (growth condition) or in RPMI (-glutamine) supplemented with 10% FBS and 1 mM sodium butyrate (differentiation medium)^49^. MCF7, CAL-51, MDA-MB-468, MDA-MB-231 and HEK293T cells were cultured in DMEM supplemented with 10% FBS. HCC1500 and HCC1806 cells were cultured in RPMI supplemented with 10% FBS. MCF10A-5E cells were cultured in DMEM/F12 supplemented with 5% horse serum, EGF (20 ng/ml), hydrocortisone (0.5 µg/ml), cholera toxin (0.1 µg/ml stock), and insulin (10 µg/ml)^35^. Cells were first transient transfected and cultured for 11 days, then the cells were seeded on the surface of matrigel (Thermo Fisher Scientific) in DMEM/F12 supplemented with 2% horse serum, EGF (2 ng/ml), hydrocortisone (0.5 µg/ml), cholera toxin (0.1 µg/ml stock), insulin (10 µg/ml), and 2% matrigel for another 11 days to form spheroids^35^. Cells were treated with MitoTracker™ Red CMXRos (100 nM) and LysoTracker™ Deep Red (50 nM) 30 min before fixation or live imaging. Bafilomycin A1 (InvivoGen) was added to cells (10 nM final concentration) 6 hours after transfection, flow cemetery was performed 18 hours later (24 h after transfection). All cell lines were transfected with Lipofectamine 3000 reagent. K562 *ANKLE1* KO lines were obtained by co-transfecting two plasmids carrying sgRNAs for *ANKLE1* or by transfecting pX458 as a control. The GFP-positive cells were sorted into 96-well plates one day after transfection. Clones were validated by PCR with the following primers: 5′- GGTTAGTCTTCCCAGGGCAC -3′ and 5′- GCCTCCCGTGTATAAGCCTC -3′ (1838 bp PCR product for WT clones vs. 270 bp PCR product for KO clones). Hemoglobin was measured by absorbance at 425 nm of ${10}^{5}$ cells suspended in 0.1 ml of PBS. For the colony formation assay, cells were treated for 24 h with 500 ng/ml Anti-Fas (human, activating) clone CH11 antibody (Milipore 05-201) and seeded on 6-well plates at the specified dilutions. In the top panel of Fig. 7g, only 10% of untreated cells were imaged to ensure that the number of colonies were visually comparable to the antibody-treated cells. Colonies were fixed with methanol and stained with Enhanced Gram Crystal Violet Ethanol Solution (ThermoFisher Scientific).

### Strep-ANKLE1 purification and DNA digestion

We transfected pStrep-ANKLE1 plasmid into HEK293T cells for 24 h and performed affinity purification MagStrep3XTBeads and BXT elution buffer (IBA Lifesciences) according to the manufacturer’s protocol. Genomic DNA and mtDNA were isolated from HEK293T cells with DNeasy Blood & Tissue Kit (Qiagen) and Mitochondrial DNA Isolation Kit (abcam). 1$\mu$g of either mitochondrial or nuclear DNA was incubated with 100 ng of Strep-ANKLE1 in a buffer containing: 20 mM HEPES-KOH pH 7.4, 2 mM MnCl2, 45 mM KCl, 50 mg/ml BSA. DNA was purified by ethanol precipitation for qPCR or directly run on 0.8% agarose gel.

### qPCR

Genomic DNA was isolated with QuickExtract™ DNA Extraction Solution. RNA isolation, cDNA synthesis, and qPCR were performed with Power SYBR™ Green Cells-to-CT™ Kit using StepOne™ Real-Time PCR System (Applied Biosystems). Mitochondria content was measured by primers specific for human mtDNA Leu(UUR) region (primers: 5′- CACCCAAGAACAGGGTTTGT -3′ and 5′- TGGCCATGGGTATGTTGTTA -3′) and genomic DNA (primers: 5′- GAGGCAGGACTCAGGACAAG -3′ and 5′- GGATGCCTCAGGGACCAG -3′). *ANKLE1* RNA level was measured by primers specific for *ANKLE1* (primers: 5′-ACACCCTTCACCAGGCAGTT -3′ and 5′- AAAACTCTGGGCCAGGAGCAA -3′) and we used the following *GAPDH* primers: 5′- TGCACCACCAACTGCTTAGC -3′ and 5′- GGCATGGACTGTGGTCATGAG -3′.

### Immunofluorescence and microscopy

Cells or spheroids were fixed with 4% PFA (20% Paraformaldehyde Solution (Electron Microscopy Science)) for 1 h. Spheroids were blocked and permabilized with 0.5% Triton, 100 mM Glycine, 10% FBS in PBS for 30 min, then stained overnight with Laminin-5 Alexa Fluor 488 Conjugated antibodies (Milipore), Cleaved Caspase-3 (Asp175) (D3E9) Alexa Fluor 467 antibodies (Cell Signaling) and ActinRed 555 (ReadyProbes, Invitrogen) overnight in 0.2% Triton, 10% FPS in PBS. Stained spheroids were mounted in ProLong Gold antifade reagent with DAPI (Invitrogen) on glass coverslips and imaged with Zeiss LSM 710 Multiphoton confocal microscope. Images were quantified using the Fiji software^50^.

### Flow cytometry

Enucleation was measured by staining with DRAQ5 (Fisher Scientific) and Phalloidin-FITC (TOCRIS) and assessing the fraction of DRAQ5 negative cells. Apoptosis was detected by staining with 7-AAD and Anexin V-PE (Apoptosis Detection Kit I - BD Pharmingen). Attune NxT flow cytometer (Life Technologies) was used for all flow cytometry experiments. For imaging flow cytometry live cells were stained with DAPI (Sigma) and run on Amnis ImageStreamX Mark II (Luminex). Cells were sorted with Influx Cell Sorter (BD Biosciences). Flow cytometry data were analysed with FCS express software.

### Western blot

Cells were lysed in IPH buffer (50 mM Tris-Cl, 0.5% NP-40%, 50 mM EDTA). Protein lysates were run on 10% polyacrylamide SDS-PAGE gels and transferred to nitrocellulose membranes (Amersham Protran 0.45 µm NC - GE Healthcare Life Sciences). Membranes were blocked for 30 min in 5% milk in TBST buffer and incubated overnight with primary antibody (1:1000). Secondary antibody (1:5000) incubation was carried out for 1 hour after washing with TBST, and before washing and incubation with SuperSignal™ West Pico PLUS Chemiluminescent Substrate (ThermoFisher). The following primary antibodies were used: Anti-Phospho-Stat1 (Tyr701) (58D6) antibody (Cell Signaling Technology 14994), Anti-Stat1 (D1K9Y) antibody (Cell Signaling Technology 80916), Anti-Phospho-Stat3 (Tyr705) (D3A7) antibody (Cell Signaling Technology 9145), Anti-Phospho-Stat5 (Tyr694) (D47E7) antibody (Cell Signaling Technology 4322), Anti-Phospho-IκBα (Ser32) (14D4) antibody (Cell Signaling 2859), Anti-Cleaved PARP (Asp214) (D64E10) antibody (Cell Signaling Technology 5625) and Anti-β-actin (AC-74) antibody (GenWay Biotech Inc. GWB-A0AC74). Densitometry was performed with Fiji^50^. Uncropped blots are included in Fig. S8.

### ATP rate assay

Percent ATP produced by mitochondria and the level of compensatory glycolysis were measured using a Seahorse XF Real-Time ATP Rate Assay Kit and XFe96 Analyzers (Agilent) according to manufacturer instructions. Seahorse XF Analyzers directly measure real time extracellular acidification rate (ECAR) and oxygen consumption rate (OCR) of cells. ECAR and OCR are indicators of the two major energy-producing pathways: glycolysis and oxidative phosphorylation. Basal OCR and ECAR rates were first measured. Injection of oligomycin results in an inhibition of mitochondrial ATP synthesis that results in a decrease in OCR, allowing for quantification of mitochondrial ATP Production Rate. ECAR data combined with the buffer factor of the assay medium allows calculation of total Proton Efflux Rate (PER) using the following formula: PER (pmol H + /min) = ECAR (mpH/min) × BF (mmol/L/pH) × Geometric Volume (μL) × Kvol. Complete inhibition of mitochondrial respiration with rotenone plus antimycin A accounts for mitochondrial-associated acidification, and when combined with PER data allows calculation of the glycolytic ATP Production Rate. Compensatory glycolysis was measured as ATP production with complete inhibition of mitochondrial respiration.

### Pulse Field Gel Electrophoresis (PFGE)

Live cells were embedded in 1% LMP agarose in 50 mM EDTA (pH 8). Solidified plugs were incubated in 1% LDS (RPI Research Products International), 100 mM EDTA, 10 mM Tris pH 8.0 overnight and then washed 5 times (30 min each wash) in 50 mM EDTA (pH 8). Plugs were then secured in 1% Certified Megabase Agarose (BIO-RAD) gel in 0.5x TBE buffer and run with Lambda Ladder (ProMega-Markers) using CHEF-MAPPER system (BIO-RAD).

### RNA-seq

Live, nonapoptotic, GFP-positive cells were sorted 1, 3, and 7 days after transfection with pEGFP-C1-ANKLE1 or pEGFP-C1 plasmids. RNA was isolated by TRIzol extraction using Direct-zol RNA MicroPrep Kit including DNase treatment (ZymoResearch). rRNA depletion was performed using a NEBNext rRNA Depletion Kit v2 (Human/Mouse/Rat) and RNA was purified using Agencourt RNAClean XP Beads (New England Biolabs). Libraries were prepared using the NEBNext Ultra II Directional RNA Library Prep Kit for Illumina (New England Biolabs). Concentrations of libraries were measured with Qubit dsDNA HS Assay Kit (Thermo Fisher Scientific) and fragment size distribution were measured with TapeStation (Agilent). Libraries were pooled together and sequenced at the University of Virginia Genome Analysis and Technology Core using the Illumina NextSeq2000 instrument. RNA-seq data were aligned to the human assembly hg38 (Gencode v33) using HISAT2^51^ and quantified by HTSeq^52^. We applied DESeq2^53^ to identify differentially expressed genes with a false discovery rate less than 0.05 (Fig. S5C). Principal component analysis was performed to ensure that replicates group together and that variation is observed between control and ANKLE1 expressing cells and time points, rather than between batches (Fig. S5A). Gene Set Enrichment Analysis (GSEA)^54^ was performed in R with the fgsea package^55^ using log_2_(Fold Change)*-log_10_(FDR) to rank all genes. Gene ontology analysis was done using Metascape^56^ on significantly (FDR < 0.05) activated or repressed genes.

### Reporting summary

Further information on research design is available in the Nature Portfolio Reporting Summary linked to this article.

## Supplementary information

Supplementary Information

Description of Additional Supplementary Files

Supplementary Data 1

Reporting Summary

## Data Availability

All RNA-seq library data files are available under GEO accession number GSE186393. Uncropped blots are presented in Fig. S8. All source data underlying the graphs and charts presented in the figures are presented in Supplementary Data File 1.
