# Supplementary material for: ANKLE1 cleaves mitochondrial DNA and contributes to cancer risk by promoting apoptosis resistance and metabolic dysregulation
Source: Commun Biol. 2023 Mar 1;6:231. doi: 10.1038/s42003-023-04611-w (PMC9977882; doi:10.1038/s42003-023-04611-w)
Supplement: Supplementary file 1 — Supplementary Information [file 42003_2023_4611_MOESM1_ESM.pdf]

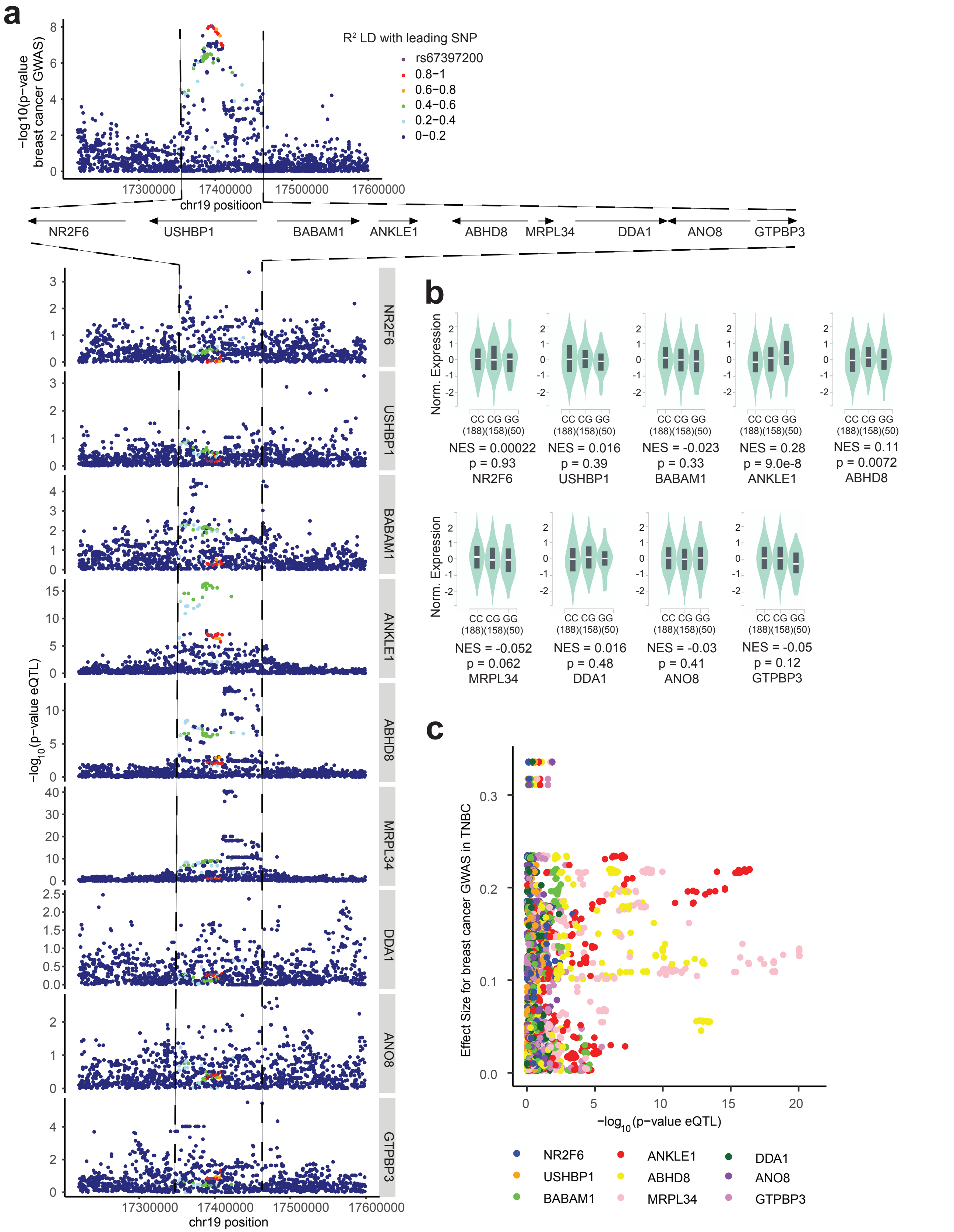

Fig. S1. Comparison of *ANKLE1* with other genes in the locus confirms that *ANKLE1* is the most likely causal breast cancer susceptibility gene within chr19p13.1. A) The breast cancer susceptibility GWAS variants within the chr19p13.1 locus (higher panel) colocalize predominantly with *ANKLE1* eQTL variants as opposed to the other local genes (lower panel). B) The G allele of rs67397200, which is associated with increased breast cancer risk, is associated with higher expression of *ANKLE1* in breast tissue. The risk allele does not associate with the expression of other genes in the locus, except *ABHD8* albeit with a less significant p-value. C) The *ANKLE1* eQTL p-value correlates best with TNBC GWAS effect size.

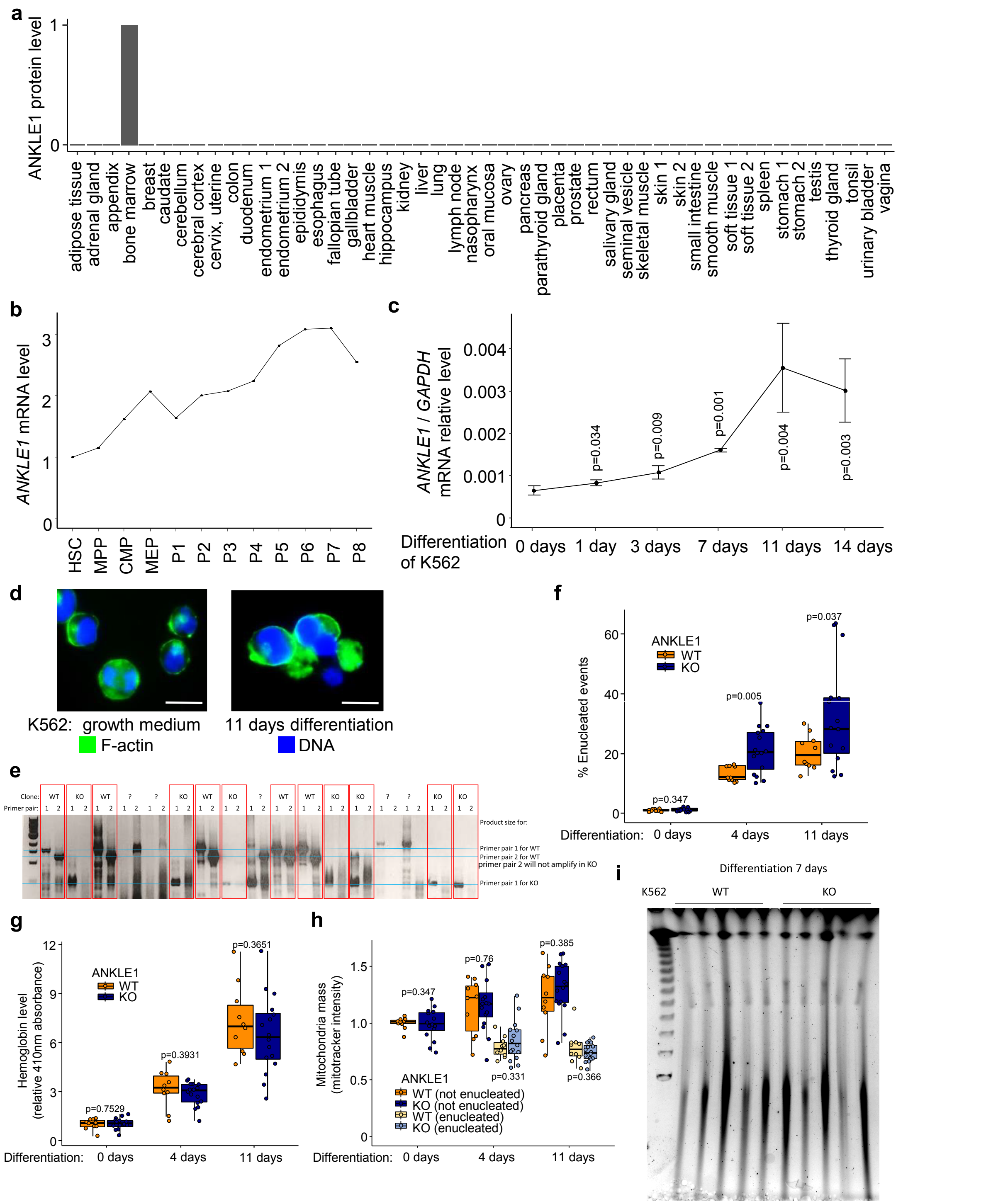

Fig. S2. ***ANKLE1* expression is only within the bone marrow and increases throughout erythropoiesis, but knockout of *ANKLE1* has only modest phenotypes.** A) ANKLE1 protein expression in human tissues is limited to bone marrow (https://www.proteinatlas.org/). B) *ANKLE1* mRNA level increases throughout erythroid differentiation (RNA-seq data: GSE115684 [21]): hematopoietic stem cells (HSC), multipotential progenitors (MPP), common myeloid progenitors (CMP), megakaryocyte erythroid progenitors (MEP), myeloid progenitor (P1) to reticulocyte (P8). C) *ANKLE1* mRNA increases in differentiating K562 cells. P-values result from a T-test compared to time point 0. D) A representative image of K562 cells shows enucleation after 11 days in differentiation medium (right panel) compared to undifferentiated cells (left panel). The scale bar is 20 µm. E) PCR validates the K562 knockout clones and tehir wild type counterparts. F) The fraction of enucleated cells is increased in *ANKLE1* KO K562 cells during differentiation. G) Hemoglobin level is unchanged upon *ANKLE1* KO in K562 cells during differentiation. H) *ANKLE1* KO does not affect mitochondria mass during K562 cell differentiation (p-values are calculated with a two-tailed t-test). I) Nuclear DNA fragmentation is no different between wild-type (WT) and knockout ANKLE1 K562 cells, as shown by pulse field gel electrophoresis analysis of DNA isolated from undifferentiated and differentiated (7 days) K562 cells.

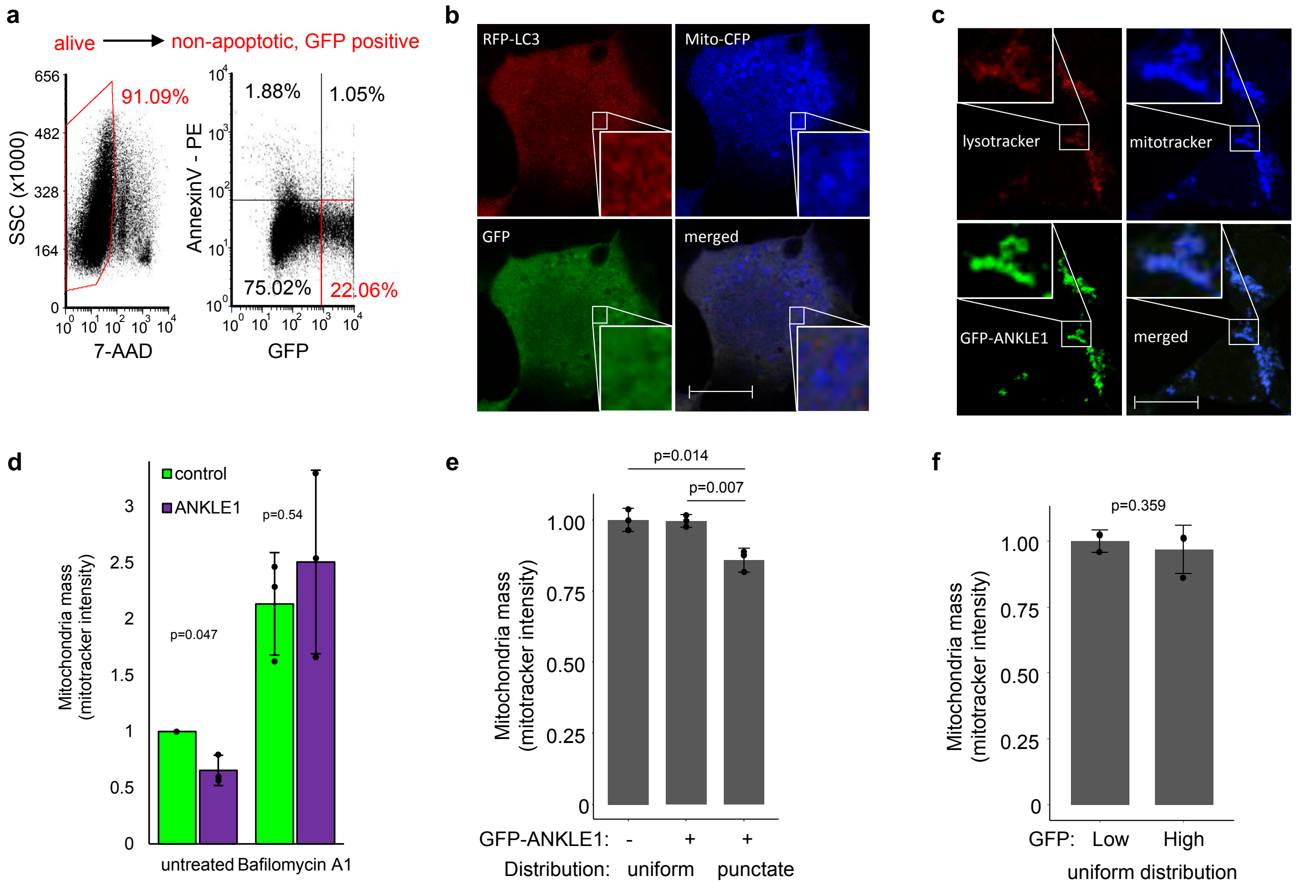

Fig. S3. *ANKLE1* expression correlates with decreased mtDNA and ANKLE1 protein localizes to the mitochondria. B) *ANKLE1* expression negatively correlates with mtDNA fold change between tumor and normal tissue in breast cancer[27]. A) Alive, non-apoptotic and GFP-positive cells were selected using the illustrated gating strategy. B) Confocal microscopy imaging (scale bar 10 µm) with fluorescent-tagged mitochondrial targeting sequence present in the N-terminus of COX8, GFP, and LC3 proteins shows uniform distribution of GFP and lack of colacalizatino between GFP, LC3, and mitochondria. This control is quantified within Figure 3C. C) Confocal microscopy images (scale bar 10 µm) of HEK293T cells overexpressing GFP-ANKLE1, stained with mitotracker and lysotracker show colocalization of ANKLE1, mitochondria, and lysosomes, as an orthogonal method to Figure 3C. D) Bafilomycin treatment abrogates the effect of ANKLE1 over-expression reducing mitochondrial mass. E) Imaging flow cytometry of HEK293T cells overexpressing GFP-ANKLE1 indicates that ANKLE1 expression causes lower mitochondria content (measured by mitotracker fluorescence) only when ANKLE1 is distributed in puncta. F) In contrast, differences in ANKLE1 intensity does not cause lower mitochondria content when ANKLE1 is uniformly distributed throughout the cell (p-values are calculated with a two-tailed t-test).

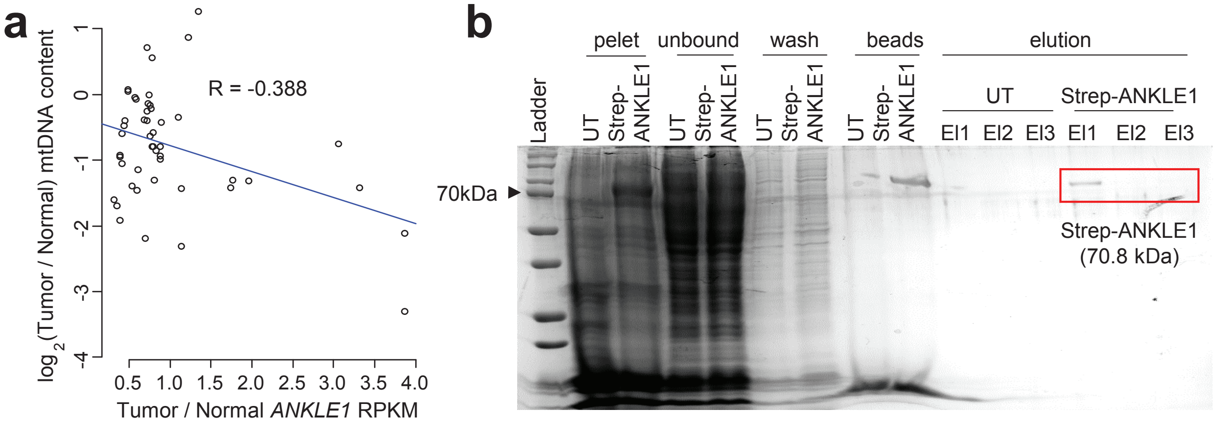

Fig. S4. ***ANKLE1*** **expression inversely correlates with mtDNA fold change between tumor and normal tissue in breast cancer.** A) The log ratio of Tumor/Normal mtDNA content on the y-axis is plotted against the log ratio of tumor/normal ANKLE1 expression on the x-axis [27]. B) We purified the ANKLE1 protein by Streptavidin-tag affinity chromatography. The UT lanes corresponds to untransfected HEK293T cell lysate purification and Strep-ANKLE1 lanes correspond to purification from HEK293T cells transfected with pStrep-ANKLE1 vector. Purified Strep-ANKLE1 is 70.8 kDa.

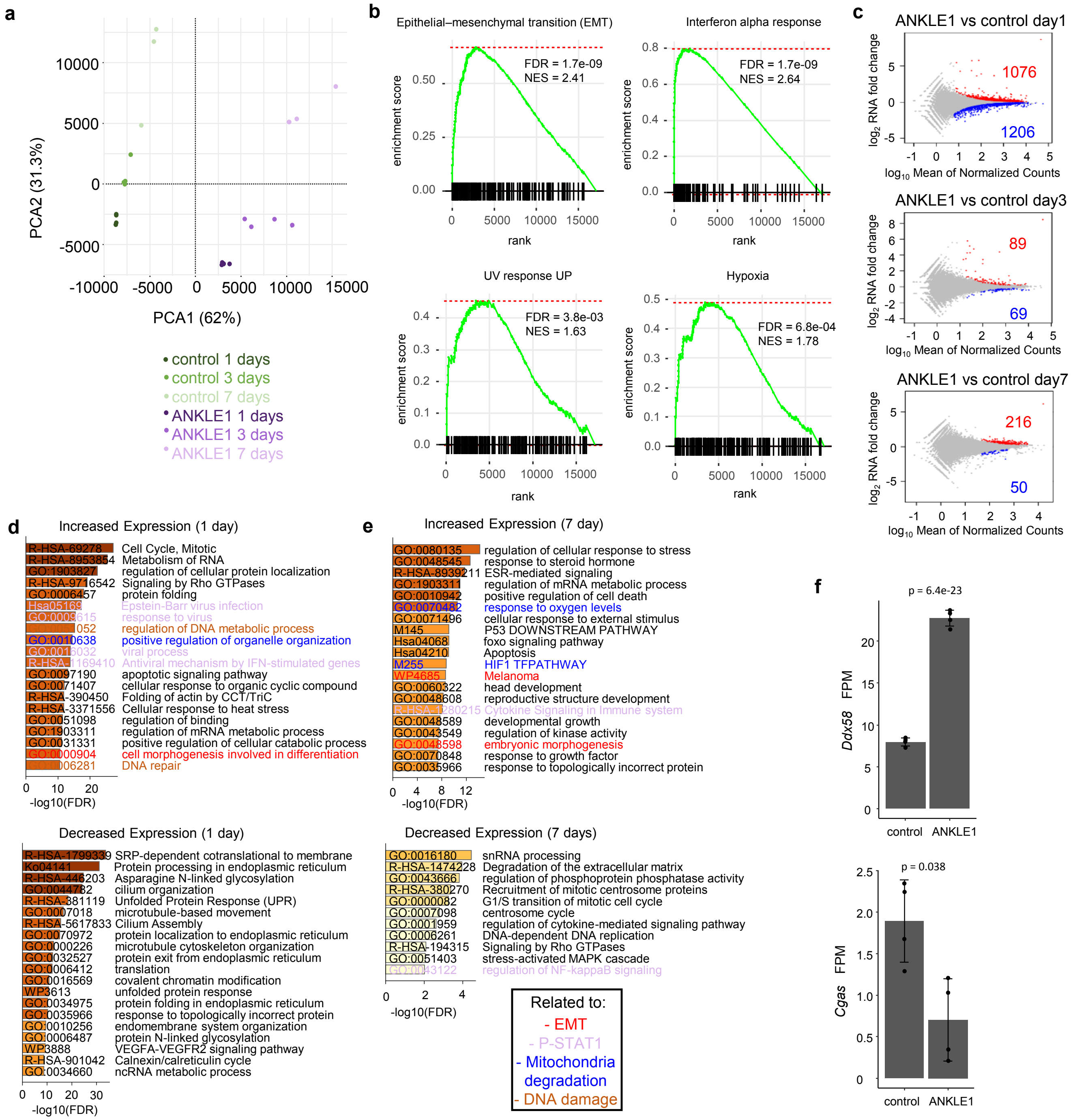

Fig. S5. RNA-seq analysis of all time points indicates that ANKLE1 expression leads to changes in EMT, STAT1 activation, mitochondria degradation, and DNA damage. A) Principal component analysis of the RNA-seq data illustrates that biological replicates cluster together. B) GSEA plots show enrichment of genes engaged in Epithelial-Mesenchymal transition, Interferon alpha response, UV response UP, and Hypoxia. C) These plots compare the change in expression of all genes upon *ANKLE1* transfection for the indicated times. Red genes are activated upon ANKLE1 expression, blue genes are repressed, and light-gray points represent all other genes. The numbers of activated and repressed genes are indicated by text with the corresponding color. D-E) Gene ontology analysis for differentially expressed genes (Figure S5C) for the 3 day (D) and 7 day (E) time points after transfection. ANKLE1 overexpressing cells versus control cells show enrichment for STAT1 activation DNA damage and mitochondria degradation. F) DDX58 senses mtRNA exposed on the mitochondrial surface after mtDNA damage. cGAS is the major innate immune sensor of pathogenic DNA and can sense nuclear DNA breaks. We observe activation of DDX58 expression and inhibition of cGAS after ANKLE1 overexpression. FDR adjusted p-values from the DESeq2 analysis are shown.

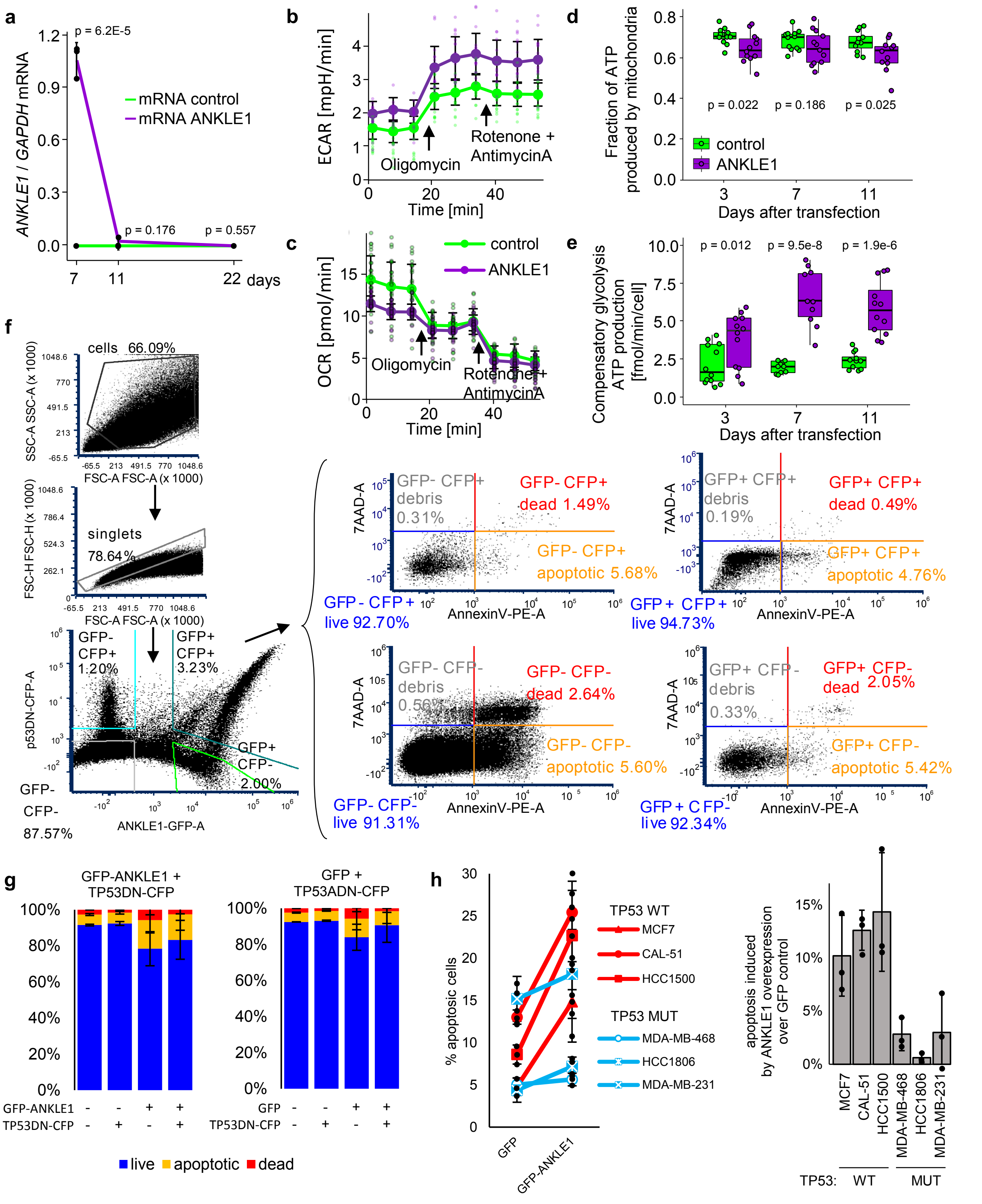

Fig. S6. ANKLE1 induces mitophagy, which results in a change in energy production and apoptosis resistance that is dependent upon TP53 inactivation. A) *ANKLE1* mRNA recovers to pre-transfection levels 11 days after ANKLE1 overexpression. B-C) ECAR (B) and OCR (C) measurements obtained during the ATP rate assay in ANKLE1-overexpressing cells show higher extracellular acidification rate and lower oxygen consumption rate compared to control GFP cells. This observation is consistent with switching of the metabolism from oxidative phosphorylation to glycolysis as a main energy source. D) ANKLE1 decreases the fraction of ATP produced by mitochondria in HEK293T cells. E) ANKLE1 increases maximum compensatory glycolysis in HEK293T (p-values are calculated with a two-tailed t-test). F) An example gating strategy isolates GFP-ANKLE1 (or control) and CFP-TP53 to assess the level of apoptosis in cells overexpressing ANKLE1 and/or a TP53 dominant negative mutant. G) The level of apoptosis in HEK293T is not affected by ANKLE1 overexpression, presumably because TP53 is already inactive in HEK293T cells. H) ANKLE1 overexpression specifically increases apoptosis in p53 wild-type cell lines (MCF7, HCC1500 and CAL51) compared to p53-mutant breast cancer cell lines (HCC1806, MDA-MB-231 and MDA-MB-468). These results support our conclusion that ANKLE1 causes apoptosis in presence of wild-type p53, and not in presence of mutant p53.

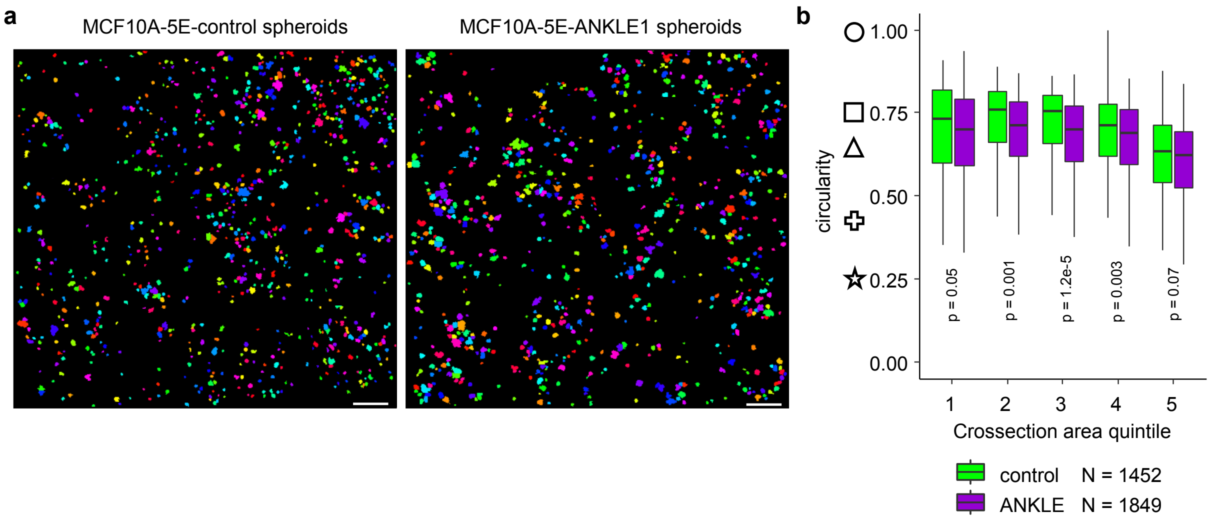

Fig. S7. ANKLE1 induces distortion of MCF10A-5E spheroids, irrespective of spheroid size. A) Images that were quantified by OrganoSeg software are distinguishable as individual spheroids. The scale bar is 500 µm. B) We separated spheroids into quintiles based upon their circularity. Note that quintiles are defined by the pooling of control and ANKLE1 cells, so the ranges of adjacent box plots are identical. These results confirm that while larger spheroids tend to be less circular, lower circularity among ANKLE1-expressing spheroids is not driven by their larger size (p-values are calculated with a two-tailed t-test).

Fig. 5D

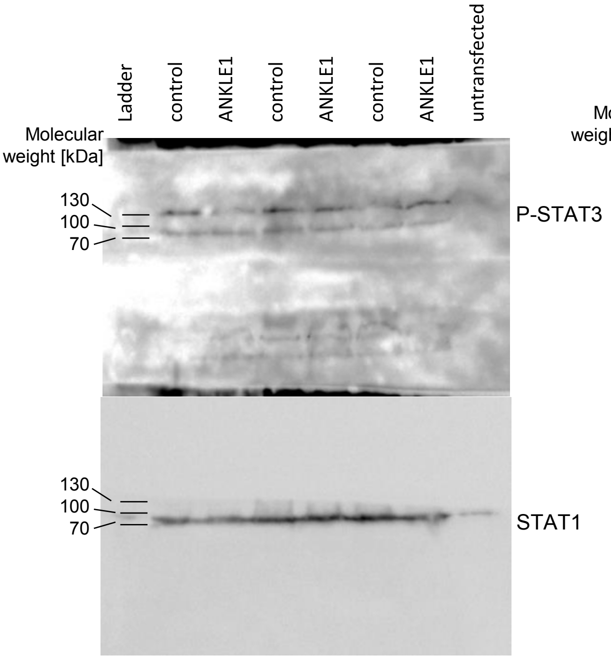

Fig. 5D

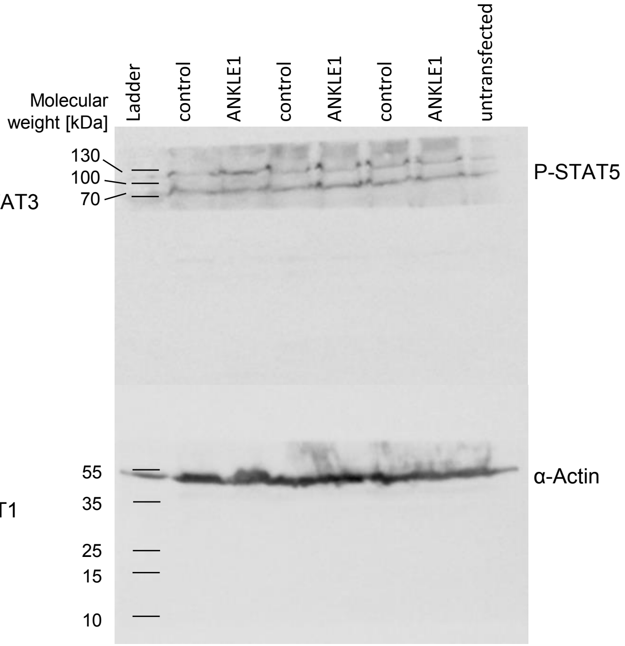

Fig. 5D

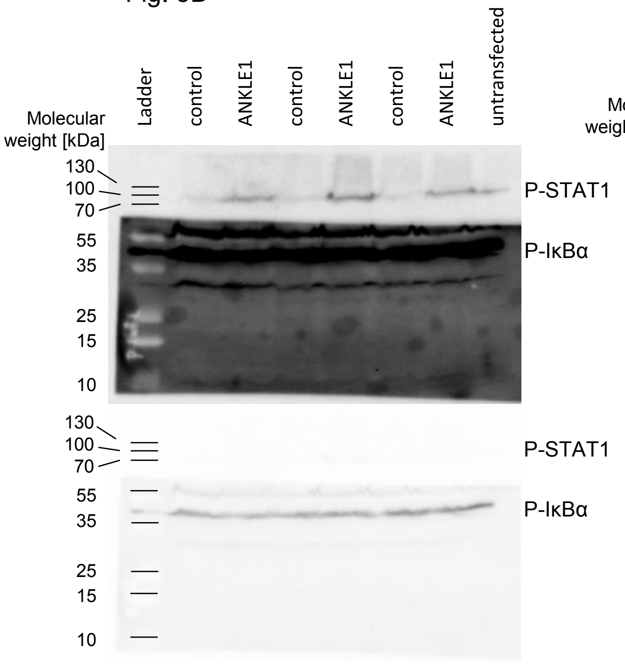

Fig. 8F

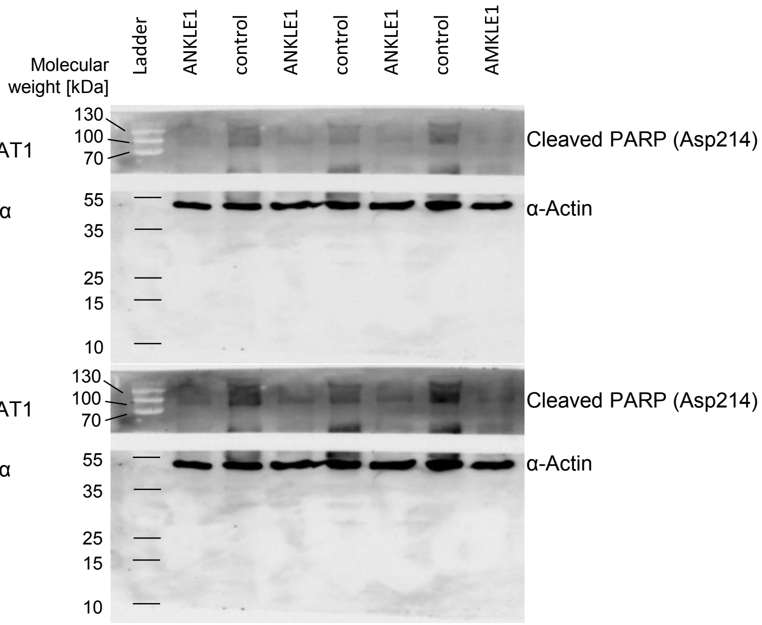

Fig. S8. The uncropped blots for the corresponding Figure panels.
