# Supplementary material for: ANKLE1 cleaves mitochondrial DNA and contributes to cancer risk by promoting apoptosis resistance and metabolic dysregulation
Source: Commun Biol. 2023 Mar 1;6:231. doi: 10.1038/s42003-023-04611-w (PMC9977882; doi:10.1038/s42003-023-04611-w)
Supplement: Supplementary file 2 — Description of Additional Supplementary Files [file 42003_2023_4611_MOESM2_ESM.pdf]

Description of Additional Supplementary Files

File name: Supplementary Data 1

Description: All source data underlying the graphs and charts presented in the figures.
